# Effects of α-conotoxin ImI on TNF-α, IL-8 and TGF-β expression by human macrophage-like cells derived from THP-1 pre-monocytic leukemic cells

**DOI:** 10.1038/s41598-017-11586-2

**Published:** 2017-10-06

**Authors:** Alberto Padilla, Patricia Keating, James X. Hartmann, Frank Marí

**Affiliations:** 1 Departments of Biomedical Sciences, Florida Atlantic University, Boca Raton, 777 Glades Rd, Boca Raton, FL 33431 USA; 2 Biological Sciences, Florida Atlantic University, Boca Raton, 777 Glades Rd, Boca Raton, FL 33431 USA; 3 Chemistry and Biochemistry, Florida Atlantic University, Boca Raton, 777 Glades Rd, Boca Raton, FL 33431 USA; 4 Marine Biochemical Sciences, Chemical Sciences Division, National Institute of Standards and Technology, 331 Fort Johnson Rd., Charleston, SC 29412 USA

## Abstract

α7 nicotinic acetylcholine receptors (nAChRs) are ubiquitous in the nervous system and ensure important neurophysiological functionality for many processes. However, they are also found in cells of the immune system, where their role has been less studied. Here we report the pro-inflammatory effect of ImI, a well characterized conotoxin that inhibits α7 nAChRs, on differentiated THP-1 pre-monocyte macrophages (MDM) obtained by phorbol 12-myristate 13 acetate (PMA) treatment. Enzyme-linked immunosorbent assay (ELISA) performed on supernatant fluids of LPS challenged MDM showed ImI-mediated upregulation of pro-inflammatory cytokine TNF-α in an ImI concentration-dependent manner from 0.5 to 5.0 µmol/L and for IL-8 up to 1.0 µmol/L. Levels of anti-inflammatory cytokine TGF-β remained practically unaffected in ImI treated MDMs. Nicotine at 10 µmol/L significantly downregulated the release of TNF-α, but showed a lesser effect on IL-8 secretion and no effect on TGF-β. Fluorescent competitive assays involving ImI, α-bungarotoxin and nicotine using MDM and the murine macrophage RAW 264.7 suggest a common binding site in the α7 receptor. This work extends the application of conotoxins as molecular probes to non-excitatory cells, such as macrophages and supports the involvement of the α7 nAChR in regulating the inflammatory response via the cholinergic anti-inflammatory pathway (CAP).

## Introduction

The α7 nAChR is a member of the nicotinic acetylcholine receptor family and is widely distributed in neuronal and non-neuronal cells. The functional α7 nAChR occurs as a homopentamer activated intrinsically by acetylcholine to mediate the intracellular flux of Na^+^, K^+^ and Ca^2+^ ions^1^. In humans, α7 is a 55 kDa highly conserved polypeptide expressed by the 10 exon CHRNA7 gene located in chromosome 15q13-14^2,3^. In leukocytes, the α7 nAChR has been reported to play a crucial role in the mechanism employed by the vagus nerve to regulate inflammation attributable to injury and infection^4^. CHRNA7 gene knock-out and vagus nerve ablation in mice exposed to endotoxin has been reported to fail to down-regulate macrophage pro-inflammatory cytokine expression in the presence of acetylcholine (ACh) while electrical stimulation down regulated production of pro-inflammatory cytokines^5–8^. Additionally, a distinct human gene dubbed CHRFAM7A, residing 1.5 Mb proximal to the 5′ end of the full length gene for α7, reveals the partial duplication of exons 5–10 from CHRNA7 and the presence of four novel exons presumably derived from chromosome 3^9,10^. The latter novel exons are transcribed in frame with the five duplicated exons in chromosome 15. The CHRFAM7A isoform runs in opposite orientation to that of the canonical CHRNA7 and it is unclear whether this duplicated isoform is translated. However, there is evidence that the CHRFAM7A is not present in every chromosome 15^10^. The existence of these two gene isoforms has been a motive of debate as to the presence, co-presence and functionality of α7 nicotinic receptors in monocytes and macrophages and their role in anti-inflammatory responses^1,11,12^.

Toxins have played a significant role in probing nAChRs and their associated cholinergic pathways in living organisms. Notably, the venom of cones snails, a genus of predatory marine mollusks, produces an extraordinary diversity of peptides (conotoxins) including several classes of compounds that target nAChRs. Conotoxins are valuable research tools as they selectively target different types of ion channels, receptors and transport molecules in neuronal and non-neuronal systems. Conotoxins have been used as molecular probes and as therapeutic leads for the nervous system and the neuromuscular junction. They show inhibitory effects in nAChRs, NMDA receptors, neurotensin receptors, norepinephrine transporters, Na^+^ channels, K^+^ channels, and Ca^2+^ channels, many of which can be involved in disease states^13,14^. One of such peptides is α-conotoxin ImI, which we used in this study to antagonize the α7 nAChR expressed in THP-1 monocyte derived macrophages and to study its corresponding inflammatory responses. ImI is a 12 amino acid peptide constrained by two disulfide bonds that inhibits α7 nAChRs. This conotoxin was originally isolated from the venom of *Conus imperialis* and it is the first α-conotoxin reported to inhibit neuronal nAChRs^15^.

The presence of nAChRs in non-excitable cells, particularly in immune cells, has been noted in the literature for a while. However, the precise role of these receptors has not been firmly defined. Findings that the efferent pathway of the vagus nerve is directly or indirectly involved in the release of ACh to modulate acute inflammation via the α7 receptor in macrophages^5,16–19^ led us to explore the effects of ImI on the α7 receptors expressed in the membranes of THP-1 MDMs. Our hypothesis was that ImI might exacerbate the pro-inflammatory effects of the LPS *E. coli* endotoxin on human macrophages by antagonizing α7 nAChRs. To this end, we demonstrated the binding of ImI to mouse and human macrophages and assessed the effect of ImI on LPS-challenged MDM by evaluating the expression levels of the pro-inflammatory cytokine TNF-α and chemotactic factor IL-8.

## Results

### Cell Viability and Proliferation Assays

**Figure 1:**
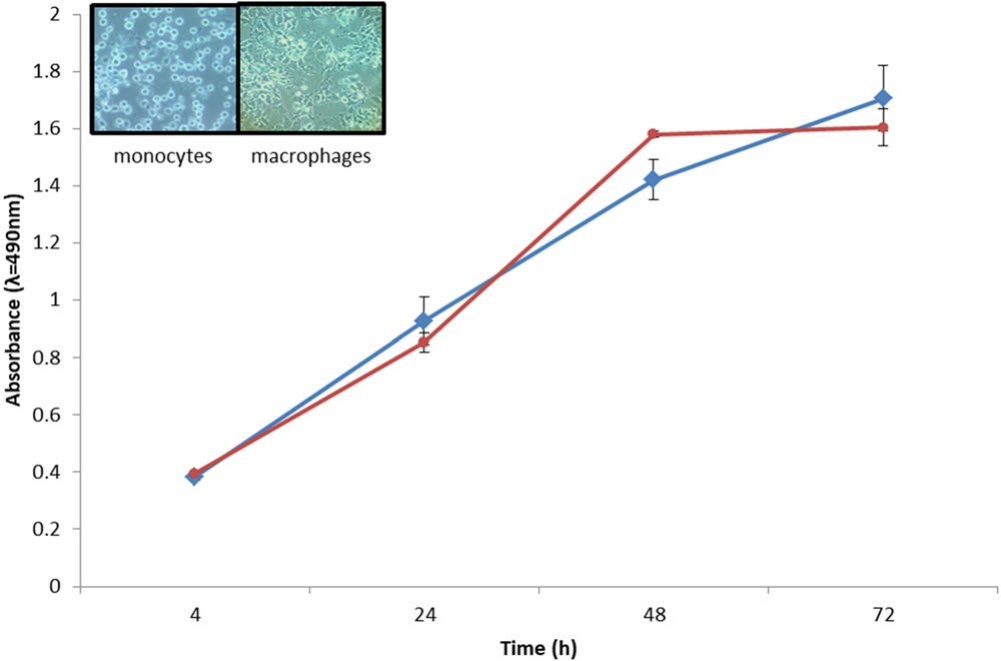
PMA induced differentiation of THP-1 monocytes into adherent macrophage-like cells was observed under the light microscope (inset). Cells with macrophage morphology ceased their proliferation after 48 h of being treated with PMA (red line) and maintained constant viability for 72 h. Untreated monocytes continued to proliferate continuously for 72 hours (blue line). Absorbance measurements are the conversion of the dye MTS used to ascertain viability and cellular proliferation.

MTS cell viability and proliferation assays conducted on untreated and PMA-treated monocytes indicated continuing viability for up to 72 h (Fig. 1). Growth stabilization in PMA-treated cells occurred after 48 h where the vast majority of cells adhered to the plate and displayed clear morphological changes under the light microscope. Morphological differentiation was initially observed after 18 h of treatment with 50 ng of PMA (see Supplementary Fig. S2). These results suggest that complete THP-1 monocyte differentiation into MDMs occurs at 48 h. This observation was used as the optimum time to select MDMs for experiments.

### Flow Cytometry

**Figure 2:**
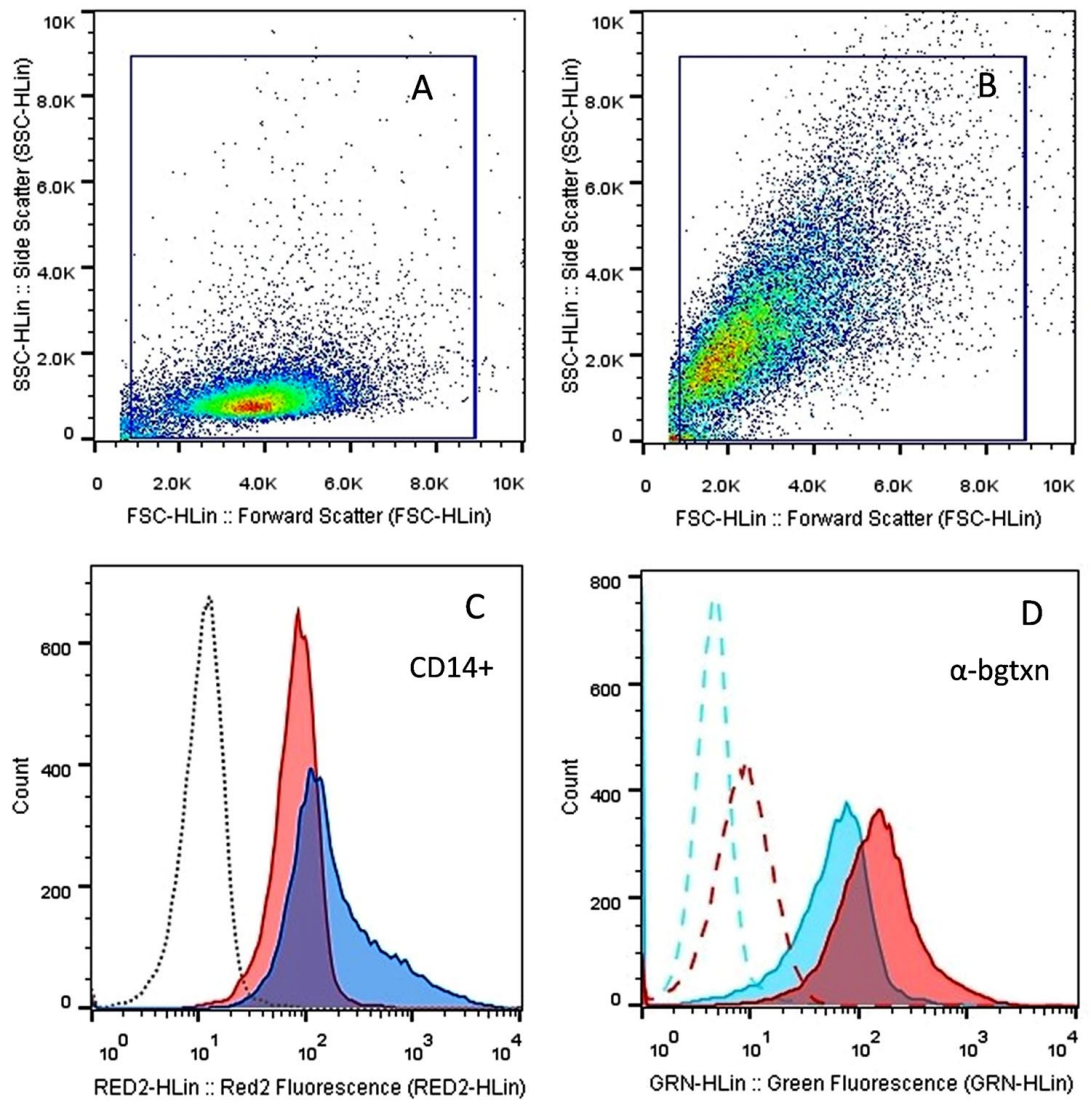
Flow cytometry dot plots representing forward scatter versus side scatter for the pre-monocytic cell population (**A**) and the PMA differentiated macrophage-like cell population (**B**) analyzed for the presence of the LPS-binding-protein receptor CD14 (**C**) and α7 nAChRs (**D**). A higher expression of CD14 receptors (MFI 182.44) is observed in PMA-derived macrophage-like cells (blue peak) while the untreated pre-monocytic cells (pink peak) show a lower fluorescent shift (MFI 82.27). CD14 receptors bind LPS-binding-protein, which in turn interacts with TLR-4 to activate cytokine expression pathways. An increased binding of fluorescently labelled α-bungarotoxin (α-BTX) (MFI 153.75) to α7 nAChRs was also observed in cells turned macrophage-like after a 48 h exposure to PMA, denoted by the pink peak (**D**). The shift for the untreated THP-1 pre-monocytic cells (MFI 64.85), represented in cyan, indicates that a lower number of α7 nAChRs are present in non-differentiated cells (**D**).

**Figure 3:**
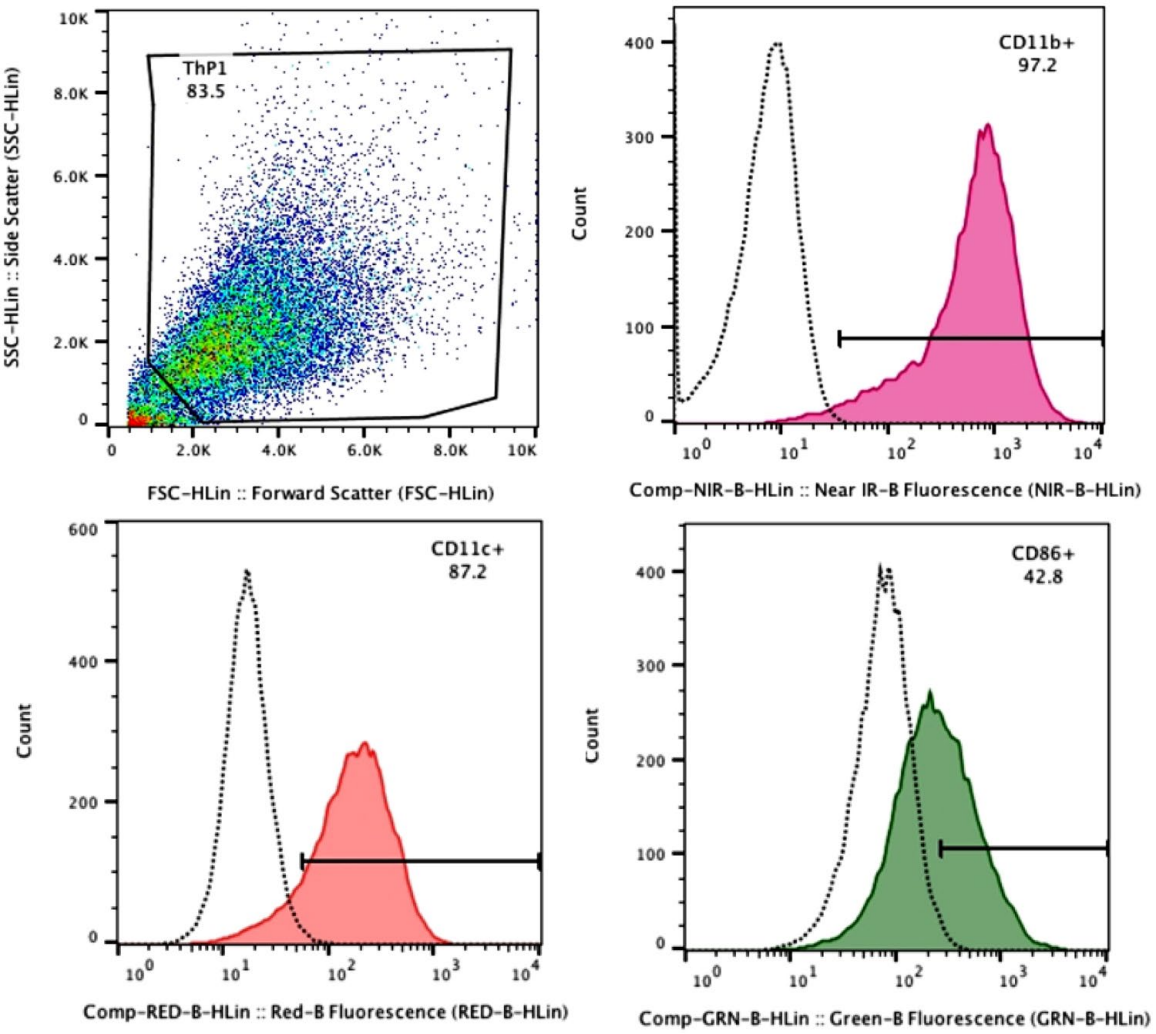
Forward scatter vs side scatter flow cytometry dot plot (top left) representing one population of PMA treated THP-1 cells assayed with anti-human monoclonal antibodies for differentiation/maturation markers CD11B (magenta peak- top right), CD11C (orange peak- bottom left) and CD86 (green peak- bottom right). The corresponding isotypes are shown in dotted lines. The expression of these markers suggests an M1 macrophage-like phenotype for the MDM populations used in this study.

Flow cytometry measurements showed a significant increase in the LPS-binding protein receptor CD14 and α7 nAChRs on rested MDM in contrast to undifferentiated THP-1 pre-monocytic cells. The difference in mean fluorescence intensity (MFI) for CD14 between pre-monocytic cells (MFI 82.27) and in rested MDM (MFI 182.44) suggests differentiation of monocyte precursor cells into MDM^20,21^ (Fig. 2A). Likewise, the MFI of α-bungarotoxin binding to the α7 nAChR was significantly higher in macrophage-like cells (MFI 153.75) than in pre-monocytic cells (MFI 64.85), which is an indication of higher expression of α7 nAChRs upon differentiation (Fig. 2B). Further assays for differentiation/maturation markers on MDM revealed the expression of CD11B, CD11C, and CD86 suggesting M1 macrophage-like phenotypic characteristics^21–26^ (Fig. 3). MDM did not express CD80, CD68 or CD163 in any of the three populations assayed (see Supplementary Fig. S4).

### Fluorescence Microscopy

**Figure 4:**
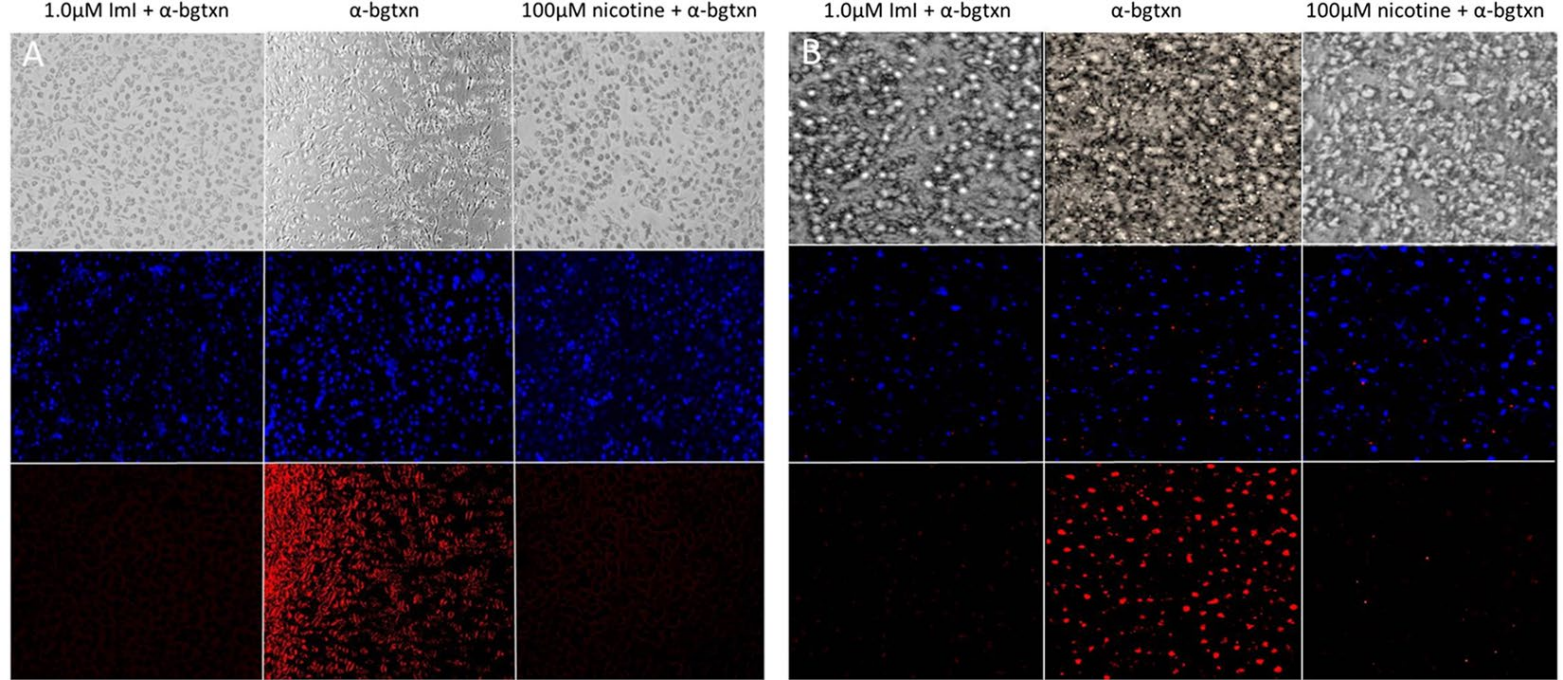
Fluorescence microscopy on human THP-1 monocyte derived macrophage-like cells (**A**) and murine macrophages RAW 264.7 (**B**). The presence of the α7 nAChRs in cells (bottom center quadrants) was observed using fluorescently labelled α-BTX. α7 competitive assays between ImI and α-BTX (bottom left quadrants) and between nicotine and α-BTX (bottom right quadrant) reveal that α-BTX, ImI and nicotine compete for the same nicotinic receptor and are likely to have a common binding site. Nuclei were labeled with Hoechst 33342 (middle rows).

To assess whether cytokine responses and nicotine modulation were attributed to interactions of ImI and nicotine with the α7 nicotinic receptors in MDM, competition assays were performed using nicotine, ImI and fluorescently labeled α-BTX. The experiments confirmed the presence of α7 receptors in MDM and murine macrophages and revealed competition of the three ligands for the same binding site in human MDM and 264.7 murine macrophages (Fig. 4A and B).

### PMA Activation

**Figure 5:**
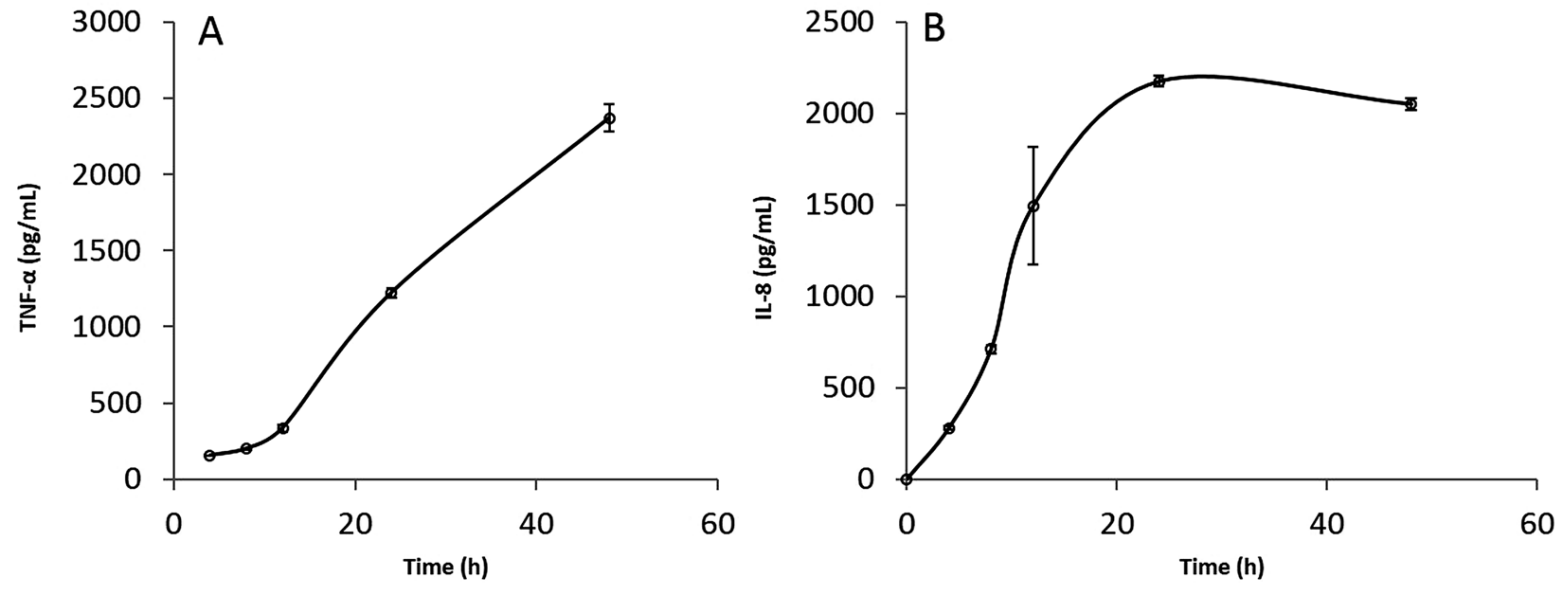
The PMA induced differentiation of THP-1 monocytes into macrophage-like cells elicits a substantial expression of TNF-α (**A**) and IL-8 (**B**) cytokines. Hence, the macrophage-like cells were “rested” for 5–7 days prior to LPS activation, in order to avoid confounding results when determining cytokine expression due solely to LPS. All measurements reflect the mean ± SEM, n = 3.

PMA alone elicited a pro-inflammatory cytokine response (Fig. 5A and B). To avoid over-estimating cytokine measurements from PMA activation, MDM were rested for 5 days, with continuous change in media, prior to treatments with LPS, ImI and nicotine.

### Time course of Optimum Cytokine Secretion from Monocytes versus MDM

**Figure 6:**
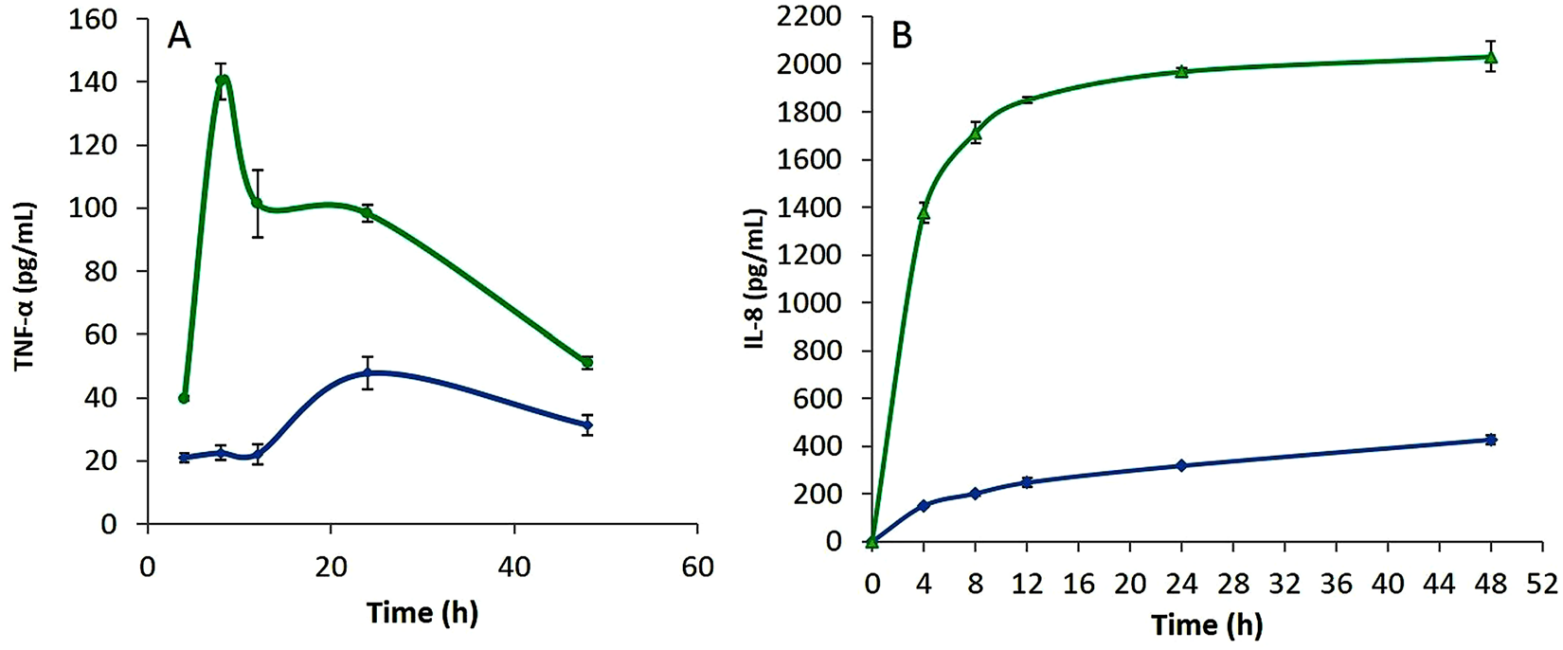
Time course and optimum expression of LPS induced TNF-α (**A**) and IL-8 (**B**) from monocytes and “rested” macrophage-like cells. THP-1 monocytes (blue) expressed significantly lower cytokine levels than monocyte-derived macrophages (MDM) (green). The highest level of TNF-α in MDM was observed at 8 h and at 48 hours for IL-8. IL-8 was collected at 24 h of LPS activation to assure steady cell viability and less stress. All measurements reflect the mean ± SEM, n = 3.

Highest expression levels of TNF-α and IL-8 from LPS activated THP-1 pre-monocytes were observed at 24 and 48 h, respectively. LPS activated MDM yielded highest expression of TNF-α in 8 h and in 48 h for IL-8 (Fig. 6A and B). Following differentiation, MDM were washed with media to remove excess PMA, and were rested for 5 days in new media before challenging with LPS. A signiwficantly higher cytokine output was obtained from MDM compared to monocytes following LPS stimulation. No significant difference in IL-8 expression was evident between 24 and 48 h (Fig. 6B). To minimize possible cell damage and cell death, subsequent IL-8 analyses were performed in MDM supernatant fluids collected at 24 h.

### Response of LPS challenged MDM to ImI and Nicotine

**Figure 7:**
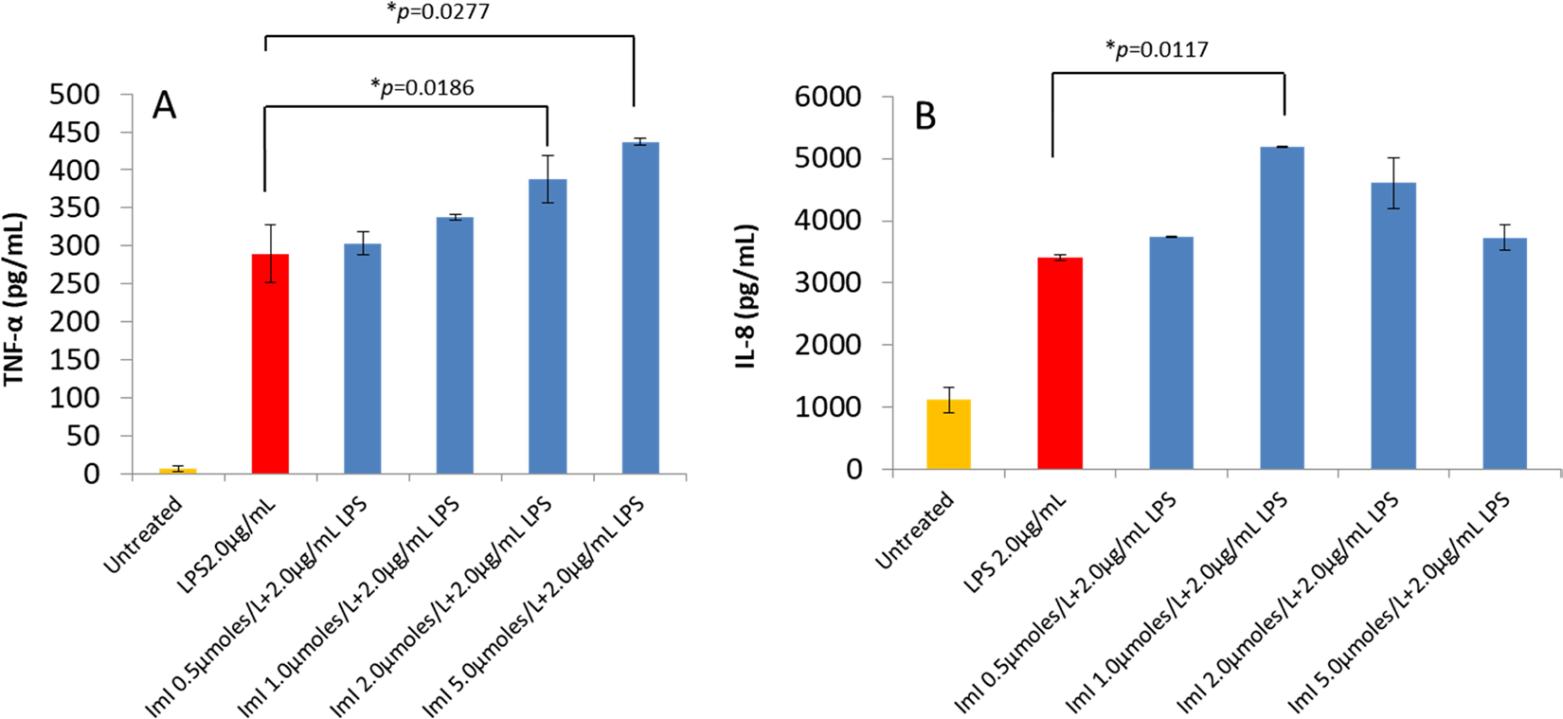
Effect of conotoxin ImI on the release of TNF-α (**A**) and IL-8 (**B**) from THP-1 derived macrophages activated with *E. coli* LPS. ImI increases the expression of TNF-α in LPS activated monocyte-derived macrophages in a concentration dependent manner from 0.5 µmol/L to 5.0 µmol/L. ImI induced cytotoxicity was observed at a concentration of 10 µmol/L (Fig. S3). IL-8 expression is likewise enhanced in the presence of ImI at 0.5 µmol/L and 1.0 µmol/L concentrations; nevertheless, downregulation of the chemokine was observed at concentrations of 2.0 µmol/L and 5.0 µmol/L. All measurements reflect the mean ± SEM, n = 3.

Rested MDM challenged with LPS for 8 h secreted significant (*p* < 0.05) TNF-α (289.59 pg/mL) versus the control group (6.51 pg/mL) (Fig. 7A). The LPS-induced secretion of IL-8 was 3402.92 pg/mL at 24 hours in comparison to 1116.70 pg/mL for the untreated control. The increasing levels of IL-8 by 24 h in the control group suggest basal constitutive production of IL-8 (Fig. 7B).

Addition of ImI (0.5 µmol/L to 5.0 µmol/L) to LPS-challenged MDM revealed a gradual increase in TNF-α (Fig. 7A). Significant (*p* < 0.05) levels of soluble TNF-α were achieved at 2.0 µmol/L ImI (387.63 pg/mL TNF-α) and 5.0 µmol/L (436.83 pg/mL) compared to the control group (289.58 pg/mL). Significant cytotoxicity was observed in cell populations treated with 10 µmol/L ImI compared to cell populations treated at 2.0 µmol/L and 5.0 µmol/L (see Supplementary Fig. S3). Hence, the level of TNF-α at the 10 µmol/L experimental condition cannot be correlated to the sole effect of ImI and, consequently, is not reported. Concentrations of ImI of 0.5 µmol/L and 1.0 µmol/L also had an upregulating effect on the expression of IL-8. Significant (*p* < 0.05) IL-8 expression was observed at 1.0 µmole/L. IL-8 began a gradual decrease in expression at 2.0 µmol/L of ImI and was stabilized at an ImI concentration of 5.0 µmol/L to nearly the same level observed when cells were stimulated with LPS alone (Fig. 7B).

**Figure 8:**
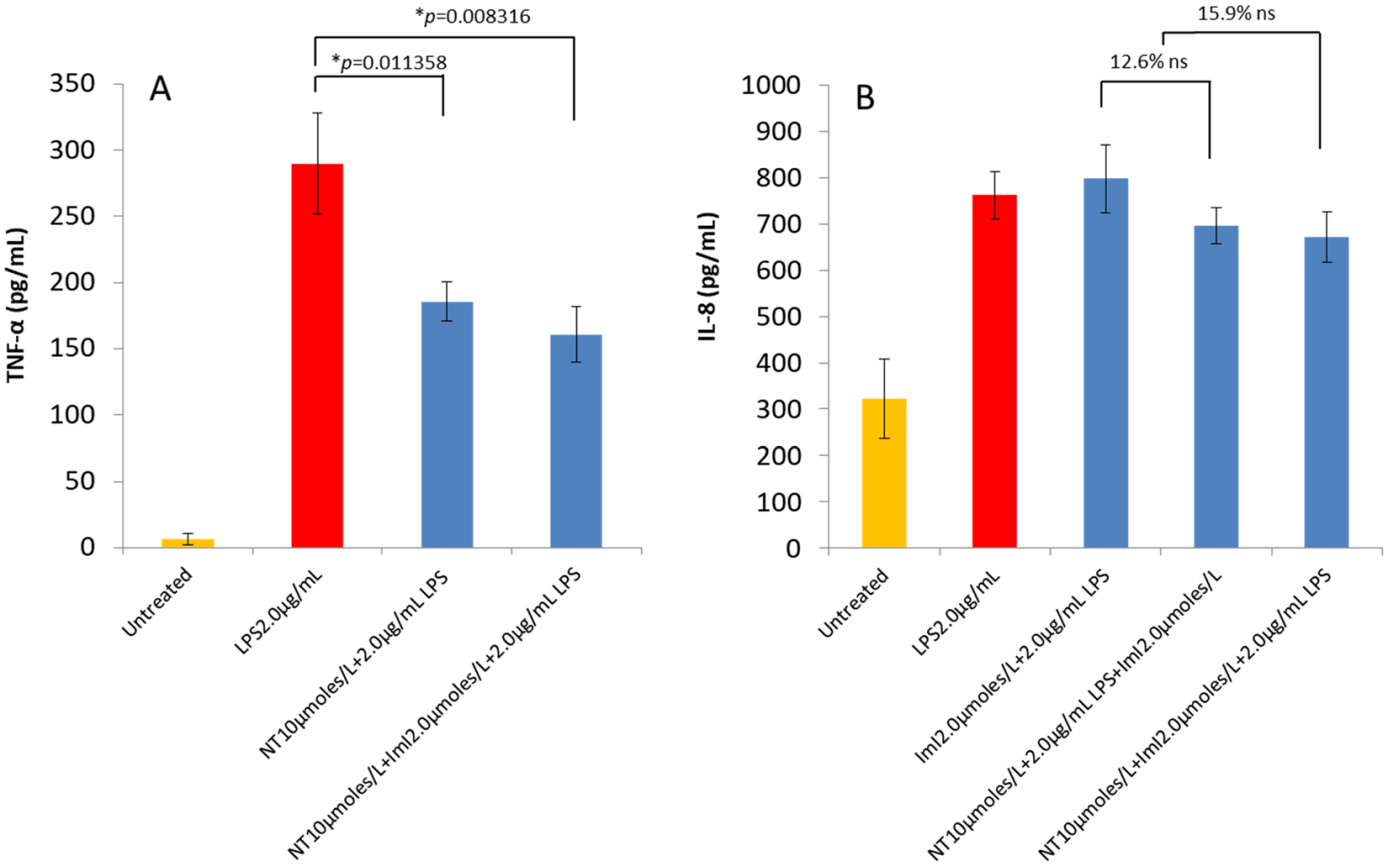
Nicotine reveals significant (*p* ≤ 0.05) modulation of TNF-α expression in monocyte derived macrophages incubated for 30 min with nicotine prior to treatments with 2.0 µmol/L ImI, 2.0 µg/mL LPS or combined (**A**). Incubation with nicotine shows a lesser (12.6–15.9%) but perceptible down regulation of IL-8 in the presence of LPS and ImI combined at 2.0 µg/mL and 2.0 µmol/L, respectively (**B**). All measurements reflect the mean ± SEM, n = 3.

Nicotine (10 µmol/L) significantly (*p* < 0.05) modulated the expression of TNF-α in LPS treated THP-1 macrophage-like cells (Fig. 8A). However, its slight modulating effect of 10–15% on IL-8 expression, gave a *p* value greater than 0.05 (Fig. 8B).

**Figure 9:**
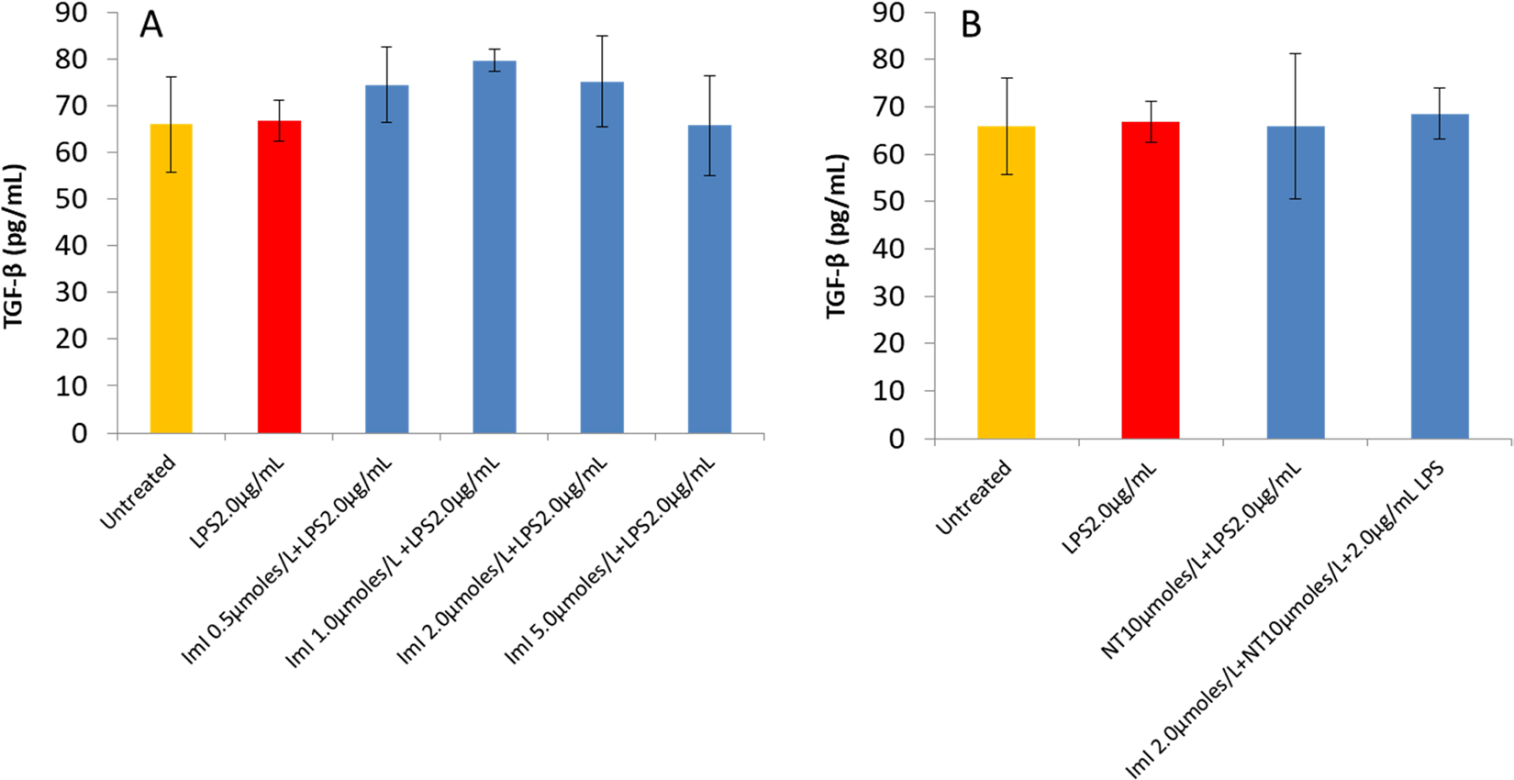
The concentration of anti-inflammatory cytokine TGF-β, in LPS activated MDM, remained at low levels and practically unchanged during increasing concentrations (0.5–5.0 µmol/L) of ImI (**A**) or during exposure to 10 µmol/L nicotine alone or in combination with ImI. ImI anti-inflammatory effects or nicotine modulatory effects were not detected. All measurements reflect the mean ± SEM, n = 3.

The levels of anti-inflammatory TGF-β remained low and practically unaffected by the increasing concentrations of ImI from 0.5 to 5.0 µmol/L (Fig. 9A) or exposure to 10 µmol/L nicotine (Fig. 9B).

## Discussion

Immune cells and neuronal cells share several identical receptors and ion channels including muscarinic and nAChRs, NMDA receptors, AMPA receptors, kinate receptors, sodium channels, potassium channels, and calcium channels^27–29^. Conotoxins have been employed in the study of these receptors within the nervous system^13^,^14,30^; therefore, conotoxins are promising molecular tools to study cellular processes and ascertain their pharmacological potential in non-excitable cells^30^. In macrophages, the α7 nAChR is a major component of the cholinergic anti-inflammatory pathway (CAP) serving as a receptor for the ACh released directly by the efferent fibers of the vagus nerve or indirectly, through vagus nerve activated T cells, to suppress inflammation provoked by tissue injury or pathogenic invasion^2,16,18,19^. Using PMA, we differentiated THP-1 pre-monocytes into macrophage-like cells and evaluated the effects of α-conotoxin ImI on the inflammatory response *in vitro*. PMA differentiates macrophages and activates both protein kinase C and NF-kB^20,31^ and during this event the levels of cytokines were highly prominent, which might indicate that NF-kB was translocating into the nucleus to upregulate the expression of TNF-α and IL-8.

Flow cytometry revealed a substantially higher expression of macrophage specific differentiation antigen CD14 receptors in PMA treated THP-1 than in the untreated monocytes. Similarly, significantly higher numbers of α7 nAChRs were found on cells treated with PMA than in the untreated THP-1 monocytes. These conditions were achieved 48 h post PMA induction with concomitant amoeboid-like cell shape and widespread plate adherence. The higher abundance of CD14 induced by PMA facilitates the docking of the lipopolysaccharide binding protein (LPS-BP) required to form a ternary complex with TLR-4 leading to activation of NF-kB and optimal cytokine expression in LPS induced events^20,32^. Together with CD14, the adequate presence of α7 receptors, required for ACh binding, was critical in our cell model to enable the quantitative evaluation of an inflammatory response in single or combined presence of LPS, ImI and nicotine. Thus, the most suitable cellular phenotypic conditions to carry out our experiments were found in 48 h PMA differentiated THP-1 cells or monocyte derived macrophages (MDM). MDM yielded from 5 to 7 fold higher cytokine expression in response to LPS challenges versus untreated THP-1 cells, confirming MDM to be a more appropriate model over undifferentiated THP-1. During PMA induced differentiation in THP-1 cells, important p38 MAPK independent mRNA stabilization takes place, significantly stronger than the mRNA stabilization induced by endotoxin^33^. To discriminate LPS induced cytokine levels in MDM from those levels initially induced by PMA, MDM were rested for 5 to 7 days with continuous change of media until cytokine expression was restored to basal levels. To ensure that cytokine response in MDM was indeed due to the primary involvement of α7 nAChRs, we ran a competition assay in presence of fluorescent α7 nAChR selective α-Bgtx, nicotine and α-conotoxin ImI. Fluorescence microscopy in MDM revealed α-Bgtx, nicotine and ImI competing for a common binding site.

In LPS-challenged MDM treated with ImI, TNF-α expression was up-regulated in a concentration dependent manner. Down-regulation of TNF-α expression was observed in MDM exposed to nicotine and further challenged with LPS. A similar regulatory effect by nicotine was observed when MDM were first exposed to nicotine before simultaneously being challenged with ImI and LPS. ImI and nicotine by themselves did not disturb MDM viability or cytokine homeostasis in absence of LPS (Figs S1 and S3). These results are in agreement with mechanisms proposed for acetylcholine modulation of pro-inflammatory cytokine levels during inflammation^2,4,5,7,34^. Additionally, the results confirm apparent selectivity, affinity and inhibitory function of α-conotoxin ImI on α7 nAChRs of human macrophages. Besides the α7 subtype, which is clearly involved in cholinergic signaling via ACh released by the vagus nerve or acetylcholine producing T cells^16,18,19^, recent *in vivo* and *in vitro* studies suggest the involvement of subtypes α5, α7, α9, β2 and β4 in the nicotine mediated mitigation of the immune response in monocytes and macrophages^35^. This evidence, should not have a significant effect in our results as α7 and α9 homopentamers appear to be the predominant functional types in cells of the immune system^36^. Nicotine was chosen as agonist, over acetylcholine, in order to bypass a sustained modulatory effect in the cell system due to the expression of acetylcholinesterase (AChE) during LPS stimulation^37^. Nicotine binding to subtypes α7 and α9 has also been determined to play important roles in cholinergic modulation of M1 monocytes^36^ and pro-inflammatory cell recruitment into the CNS which can translate into protection toward encephalomyelitis^35,38,39^. Hence, besides α7, a series of other nicotine targeted AChR receptors are apparently implicated in the complex process of an immune response. Further studies are necessary to ascertain the nAChR subtype combinations, present in macrophages, which are specifically involved in the CAP, in addition to the clearly defined α7 homopentamer.

LPS challenged MDM produced levels of IL-8 chemokine nearly 14 times higher than the levels observed for TNF-α. Moreover, IL-8 was constitutively expressed and longer-lived than TNF-α. In macrophages, the short half-life of TNF-α is due to transcriptional and post-transcriptional regulation; its mRNA remains unstable and hypoadenylated upon stimulation of macrophages by lipopolysaccharides^40^. The high levels of IL-8, in turn, are due to de-repression of the AU rich elements (ARE) found in the mRNA of most cytokines, including IL-8^33^. Additionally, the presence of TNF-α exacerbates the expression of IL-8 in macrophages^41^. In one study, TNF-α’s enhancement of IL-8 expression was reported to be at least 100 fold^42^.

IL-8 levels for MDM treated with ImI in the presence of LPS peaked significantly at 1.0 µmol/L and showed a decline at concentrations higher than 1.0 µmol/L (Fig. 7B). ImI and nicotine by themselves did not affect cytokine levels (Fig. S1). IL-8 characteristically shows variation in its expression levels, in part, as a consequence of its need to synchronize several conserved transcriptional and post-transcriptional converging regulatory pathways involving NFk-B, JNK and p38 MAPK to activate and maximize expression^42^. However, further studies are necessary to identify the cause for the subsiding levels of IL-8 at higher concentrations of ImI. Among the possibilities are the desensitization of α7 at levels above 1.0 µmol/L ImI and activation of cellular mechanisms repressing IL-8 promotion factor (NKRF) present in THP-1 cells^43^. More details on IL-8 gene activation/inhibition, signal transduction pathways and molecular functionality of the α7 nAChR and its CHRFAM7A isoform in macrophages are necessary to assess this effect. The modulating effect of nicotine, although subtle, was overpowered by high levels of IL-8 (Fig. 8B).

TGF-β is commonly expressed by M2 macrophages in anti-inflammatory environments where wound healing and tumor progression are favored. For these processes, the levels of TNF-α and other pro-inflammatory cytokines are downregulated below the threshold of inflammation^44,45^. Here we observed low levels of TGF-β and upregulation of the pro-inflammatory cytokines TNF-α and IL-8 in LPS activated MDM exposed to increasing concentrations of ImI. This, together with the differentiation/maturation markers observed in the PMA treated THP-1 pre-monocytic cells, corroborates that the type of cells used in our model to study the effects of ImI in the α7 nAChRs have a macrophage-like phenotype.

The effect of ImI in this *in vitro* cellular study provides further evidence for involvement of the α7 nAChRs in the modulation of pro-inflammatory cytokines mediated by the direct or indirect release of ACh from the efferent fibers of the vagus nerve^2,5,6,16,18,19^. Contrary to the nervous system, recent studies on the α7 in leukocytes have reported weak ionotropic functionality^46^. Evidence of alternate functionalities of α7 receptors has been provided by a novel heterotrimeric G-protein-mediated metabotropic signaling pathway in non-excitatory cells^47^. It is yet to be determined whether ACh pro-inflammatory cytokine modulation is solely due to the intervention of the canonical isoform of α7, since substantial mRNA from the hybrid gene transcript, dupα7, has been found in human macrophages acting as a dominant negative regulator of α7 nAChR *in vitro* ^48^. Furthermore, dupα7 was down-regulated by one-half in the presence of LPS, nicotine and IL-1β; thus, increasing the availability of functional α7 nAChR to attenuate the inflammatory response^48^.

The importance of the α7 nAChR in non-excitable cells and their modulation by ImI might have therapeutic applications. Observations in α7^-/-^ mouse knockouts confirmed improved antibacterial defense during *E. coli* peritonitis^49^. Additionally, abundant expression of α7 receptors leading to proliferation, metastasis and drug resistance has been reported in gastric, breast and lung cancer epithelia^50–52^. Therefore, α7 nAChRs may serve as potential therapeutic targets for the treatment of these cancer types while ImI may serve as a potential therapeutic affecting the cellular effects mediated by the α7 receptor in non-excitatory cells. Also noteworthy is the intrinsic potential therapeutic value of ImI as it does augment the levels of TNF-α and IL-8 by antagonizing the α7 nAChR. TNF-α and IL-8 have been found to be pivotal in overcoming infection by *Mycobacterium tuberculosis*, enabling macrophages to form granulomas. Direct interaction of IL-8 with erythrocytes and the bacilli itself may enhance the innate immune response^43,53,54^. A clinical study shows TB survivors having considerably higher levels of IL-8 than people who have died from the disease^53^. Therefore, the effects of ImI on the α7 nAChR could also contribute to the better understanding of the molecular aspects of TB and provide useful knowledge for the use and design of novel therapeutic agents.

This study broadens the application of conotoxins as molecular probes to explore receptors present in non-excitable systems, such as the innate immune system. The paradigm shift here proposed is to rely on the conotoxins’ consistent attributes of high affinity and specificity for receptors, ion channels and transport molecules, initially observed in the nervous system, to recognize receptors in non-excitatory cells; thus expanding the pharmacological potential for these compounds in manners unexplored to date.

## Methods

### Cell lines and cultures

THP-1 (Human Acute Monocytic Leukemia) and RAW 264.7 (murine macrophage) cell lines were kindly donated by Dr. Yoshimi Shibata, Department of Biomedical Sciences, Florida Atlantic University. Cells were maintained in log phase growth in 75 cm^2^ Falcon cell culture flaks containing complete medium (CM):Gibco RPMI 1640-L-Glutamine medium (Life Technologies- Grand Island, NY, USA) supplemented with 10% fetal bovine serum (Life Technologies- Grand Island, NY, USA) and 100 U/mL Gibco Pen Strep (Life Technologies- Grand Island, NY, USA). Cell cultures were incubated at 37°C under 5% CO_2_ in a humidified atmosphere.

### Effect of ImI and nicotine on inflammatory responses of monocytic-derived macrophage

THP-1 cells were seeded in 96 well plates with 200 µL CM containing 2.0 × 10^^4^ cells and treated with 50 ng/mL PMA for 48 h to induce differentiation into MDM, then washed 4X with CM and allowed to rest for 5 to 7 days^43^ with media exchanged for fresh media every two days. MDM were stimulated with 2 µg/mL LPS (*E. coli* serotype 055:B5 Ready-Made LPS solution −1mg/mL, 0.2µmol/L filtered- Sigma-Aldrich -St. Louis, MO, USA). ImI 0.5–5.0 µmol/L (R&D Systems Inc- Minneapolis, MN, USA) or (−)-nicotine hydrogen tartrate 10 µmol/L (Sigma-Aldrich -St. Louis, MO, USA) were added to the LPS treated MDM. Supernatant fluids were collected every 4 h for 48 h, kept frozen at −80°C, and assayed in triplicate to determine optimum expression time for LPS induced TNF-α and IL-8. The optimum expression times were observed when measuring the anti- or pro-inflammatory effects of added ImI or nicotine. TGF-β was assayed on supernatants collected at the same time interval as in IL-8. Endotoxin-free levels for ImI were confirmed using the kinetic turbidimetric method (Charles Rivers Laboratories- Charleston, SC, USA).

### Cytotoxicity of ImI toward MDM

ImI (0.5–10 µmol/L range of concentrations) were added to MDM (1 × 10^4^ cells/well with 200 µl of CM) in 96 well plates. After 24 hours of incubation, 20 µL of Cell titer 96 AQueous One Solution MTS −5 mg/mL (Promega- Madison, WI, USA) was added and incubated for an additional 4 h. Dual absorbance readings were taken in an Epoch microplate reader at λ = 490 nm and 650 nm respectively.

### Cytokine assays

Cytokines in culture supernatants fluids were assayed using complete TNF-α and IL-8 capture antibody Novex ELISA kits (Thermo Fisher Scientific, USA) according to manufacturers’ instructions. Cytokine concentrations were determined within 30 min of the stop reaction. Dual absorbance readings were taken in an Epoch microplate reader at λ = 450 nm and 650 nm respectively.

### Flow cytometry

A Guava easyCyte flow cytometer with InCyte software (EMD Millipore, USA) was used to validate both the presence of the LPS-binding-protein receptor (CD14) and the presence of α7 nAChRs. MDM cells were incubated for 1 h at 37 °C with Alexa Fluor 488 conjugated α- bungarotoxin (Life Technologies, Grand Island, NY, USA) prepared to 1 mg/mL in distilled water. Alexa Fluor 647 Anti-CD14 antibody (Imgenex, San Diego, CA), was used according to manufacturer’s directions. To determine the phenotypic characteristics of the rested MDM, cells were assayed, in triplicate, using conjugated anti-human monoclonal antibodies (mAbs) CD11b-PE-Cy7, CD11c PerCP-Cy5.5, CD80-FITC, and CD86-BB515, CD163-PE and CD68-Alexa647 (BD Biosciences, San Diego, CA). MDM were stained with mAbs for 30 minutes at 4 °C, according to the manufacturer’s protocol, and washed twice in PBS buffer prior to measuring marker expression by flow cytometry. Prior to antibody staining, MDM F_c_ receptors were blocked with anti-F_c_ antibody (BD Biosciences, San Diego, CA) according to manufacturer’s suggestion. Isotype controls (BD Biosciences, San Diego, CA) suggested by the manufacturer for each monoclonal antibody were used to calculate the change in mean fluorescence intensities.

### Fluorescence microscopy

MDM and Raw 264.7 were incubated with nicotine (100µmol/L) or ImI (1µmol/L) in 24 well plates for 16 h at 37 °C prior to the addition of fluorescent Alexa Fluor 594 α-bungarotoxin (Life Technologies, Grand Island, NY, USA) and Hoechst 33342 (Life Technologies, Grand Island, NY, USA) for 1 h at 37 °C. Cells were washed three times in PBS prior to microscopic observations. An inverted epifluorescence Accu-Scope EXI-310 with Scope LED fluorescence illuminator (Commack, NY, USA) was used to visualize the presence of nuclei and α7 nAChR in cells during competition assays. An adapted MiniVid 5.1 MP camera and ToupView micro-image analysis software (LW Scientific Lawrenceville, GA, USA) was used to capture photographs.

### Statistical analysis

Statistical analysis was performed using a two-tailed t-test where indicated. *p* < 0.05 was considered significant. Experiments and measurements were performed in triplicate.

### Disclaimer

Certain commercial equipment, instruments, or materials are identified in this paper in order to specify the experimental procedure adequately. Such identification is not intended to imply recommendation or endorsement by the National Institute of Standards and Technology, nor is it intended to imply that the materials or equipment identified are necessarily the best available for the purpose.

## Electronic supplementary material

Supplementary Information
