# Supplementary material for: Effects of α-conotoxin ImI on TNF-α, IL-8 and TGF-β expression by human macrophage-like cells derived from THP-1 pre-monocytic leukemic cells
Source: Sci Rep. 2017 Oct 6;7:12742. doi: 10.1038/s41598-017-11586-2 (PMC5630575; doi:10.1038/s41598-017-11586-2)
Supplement: Supplementary file 1 — Supplementary Information [file 41598_2017_11586_MOESM1_ESM.doc]

**SUPPLEMENTARY**

**Effects of α-conotoxin ImI on TNF-α, IL-8 and TGF-β expression by human macrophage-like cells derived from THP-1 pre-monocytic leukemic cells**

**Alberto Padilla^1,2,3^, Patricia Keating^2^, Frank Marí^3,4^ and James X. Hartmann^2^**

^1^Department of Biomedical Sciences, Florida Atlantic University, Boca Raton, FL, 33431, USA

^2^Department of Biological Sciences, Florida Atlantic University, Boca Raton, FL, 33431, USA

^3^Department of Chemistry and Biochemistry^3^, Florida Atlantic University, Boca Raton, FL, 33431, USA

^4^Marine Biochemical Science Group, Hollings Marine Laboratory, National Institute of Standards and Technology, Charleston, SC, 29412, USA


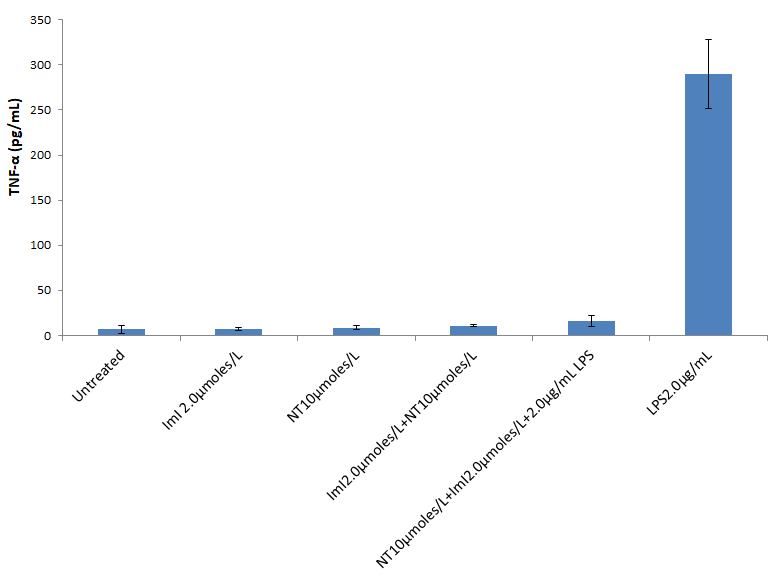


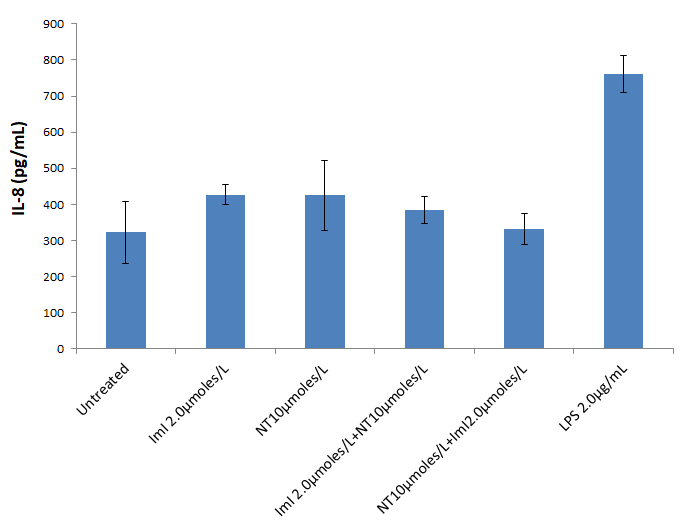


Figure S1. No significant effect, at optimum expression times of 8h and 24 h respectively, was observed for TNF-α (top) or IL-8 (bottom) cytokines on macrophage-like cells exposed to 2.0 µmoles/L ImI or 10 µmoles/L nicotine.


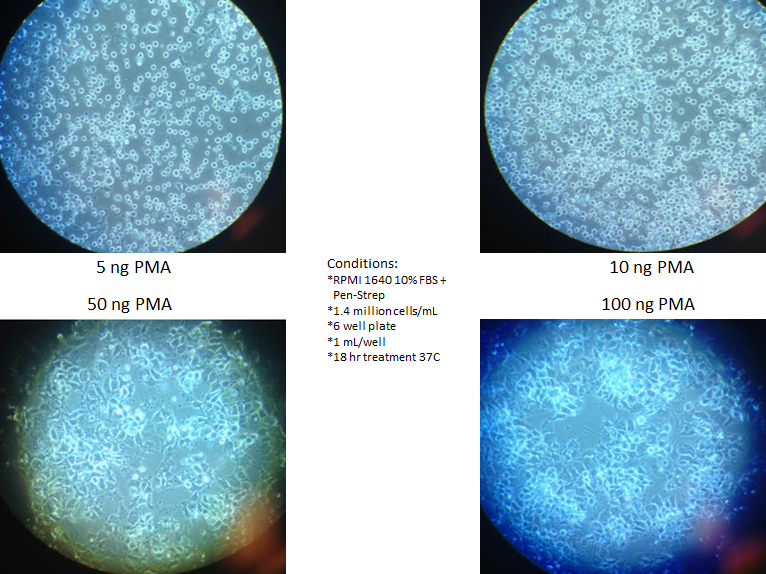


Figure S2. Suitable differentiation of THP-1 pre-monocytes occurred at PMA concentrations of 50 ng. Morphological differentiation was clearly observed after 18 h exposure to PMA.


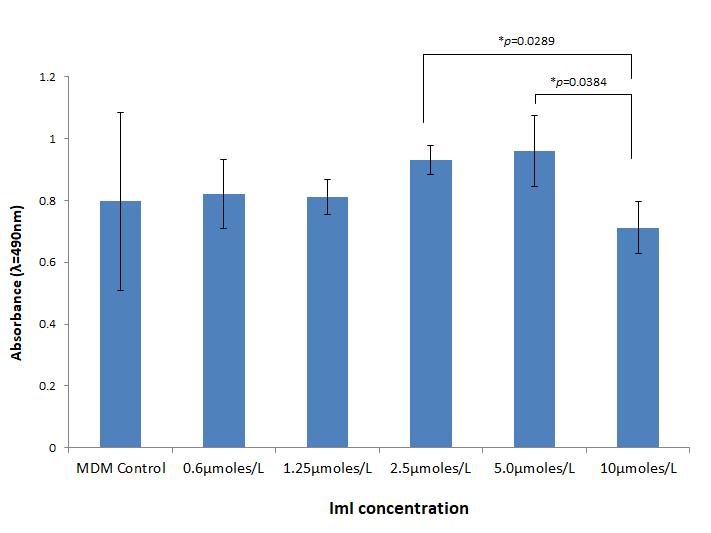


Figure S3. No cytotoxicity was observed in macrophage-like cells exposed for 24 h to ImI experimental concentrations between 0.5 and 2.0 µmoles/L. A significant (p<0.05) decrease in cell density was observed in cell populations treated at ImI concentrations of 10 µmoles/L in comparison to cell populations treated with 2.5 and 5.0 µmoles/L ImI. Hence, cytokine levels were not reported at 10 µmoles/L ImI.


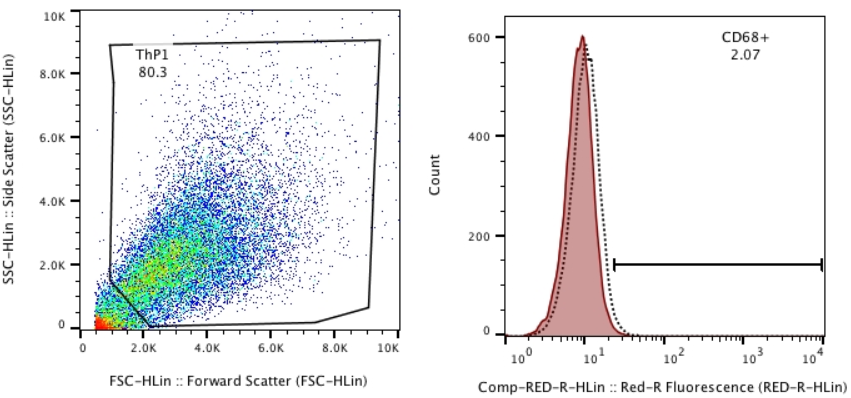


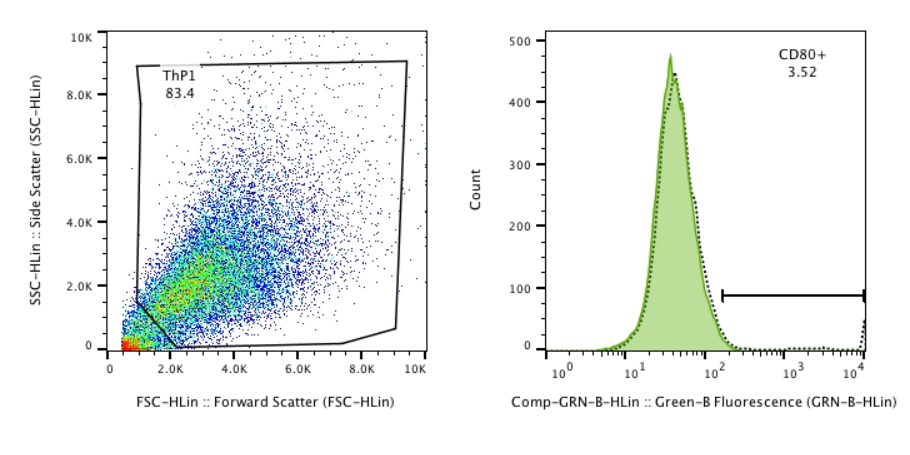

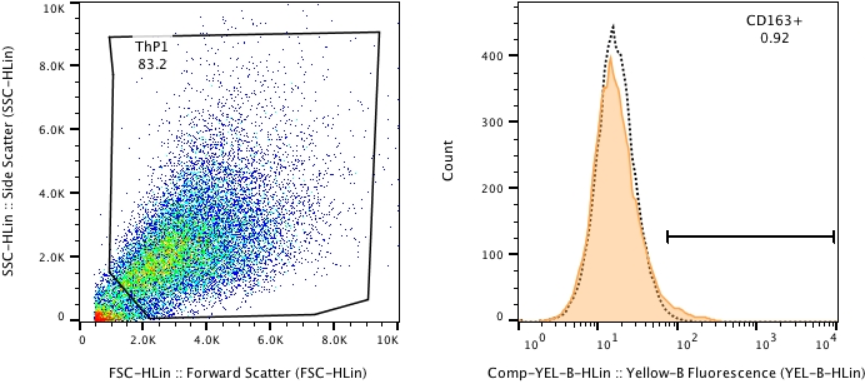


Figure S4. Pre-monocytic THP-1 cells exposed to PMA for 48 h and rested for 5 days after treatment did not express maturation/differentiation markers CD68, CD80 or CD163 (top-bottom). The differentiated cells did express CD11b, CD11c and to a lesser degree CD86. This phenotype was characteristic of the cells used in our study to measure cytokine expression.
